# Centrifugal Microfluidic System for Nucleic Acid Amplification and Detection

**DOI:** 10.3390/s151127954

**Published:** 2015-11-04

**Authors:** Baogang Miao, Niancai Peng, Lei Li, Zheng Li, Fei Hu, Zengming Zhang, Chaohui Wang

**Affiliations:** State Key Laboratory for Manufacturing Systems Engineering, Xi’an Jiaotong University, Xi’an 710054, China; E-Mails: miaobaogang@126.com (B.M.); lileixjtu@stu.xjtu.edu.cn (L.L.); horly@163.com (Z.L.); hufei0701@yeah.net (F.H.); zhangzengming@stu.xjtu.edu.cn (Z.Z.); chhw@mail.xjtu.edu.cn (C.W.)

**Keywords:** centrifugal microfluidics, PCR, real-time fluorescence detection, Lab-on-a-disc

## Abstract

We report here the development of a rapid PCR microfluidic system comprising a double-shaft turntable and centrifugal-based disc that rapidly drives the PCR mixture between chambers set at different temperatures, and the bidirectional flow improved the space utilization of the disc. Three heating resistors and thermistors maintained uniform, specific temperatures for the denaturation, annealing, and extension steps of the PCR. Infrared imaging showed that there was little thermal interference between reaction chambers; the system enabled the cycle number and reaction time of each step to be independently adjusted. To validate the function and efficiency of the centrifugal microfluidic system, a 350-base pair target gene from the hepatitis B virus was amplified and quantitated by fluorescence detection. By optimizing the cycling parameters, the reaction time was reduced to 32 min as compared to 120 min for a commercial PCR machine. DNA samples with concentrations ranging from 10 to 10^6^ copies/mL could be quantitatively analyzed using this system. This centrifugal-based microfluidic platform is a useful system and possesses industrialization potential that can be used for portable diagnostics.

## 1. Introduction

PCR is a common analytical technique in modern research and clinical laboratories. Microfluidic-based PCR has attracted attention over the last decade owing to its comparative advantages, such as a shorter assay time [1], low reagent volume and power consumption [2], high integration [3], and portability [4]. 

Currently used PCR microdevices can be classified into two major categories based on the method used to control temperatures—*i.e*., stationary or dynamic mode devices. Stationary PCR microdevices [5,6] achieve thermal cycling by alternately heating and cooling the reaction mixture in a single chamber, resulting in a relatively long reaction time. On the contrary, in dynamic microdevices, the PCR mixture flows continuously through a microchannel incorporated into two or three areas that are maintained at specific temperatures; spatially and temporally segregating the individual steps in the thermal cycling process in this manner enables more rapid amplification [7,8]. This process—known as continuous flow PCR—often makes use of a pump to move liquid through a serpentine channel [9,10,11,12]. However, pumps and linear actuators cannot be miniaturized nor can they be replaced, yielding a large instrument. Furthermore, the formation of bubbles is still a serious problem in continuous microfluidic devices, especially when an injection pump and optical detection system are used [13].

Centrifugal microfluidic systems—also known as lab-on-a-CD—are of great interest because the features required for fluidic control can be integrated into a disc, making it possible to handle fluids with only a motor [14,15]. The centrifugal microfluidic platform has been used for biomedical diagnostics, including sample preparation, immunoassays, biochemical analysis, and PCR amplification [14,15,16,17]. The centrifugal microfluidic platform has a number of advantages, such as convenient fluidic control without valves [18], an embedded fluidic pump (with centrifugal force generated by rotation of the motor), ease of multiplexing, and compatibility with many different types of liquid [14].

Centrifugal microfluidic systems that include continuous flow PCR amplification have benefits associated with miniaturization, such as reduced reagent use and improved heating rates. The centrifugal disc has been a model for microfluidic PCR amplification. Bidirectional flow control has been achieved by an inert mechanical structure that integrates pumping at different densities and power coupling in the spinning disc to achieve real-time PCR [19]. Other researcher have developed a centrifugal microfluidic system for rapid PCR amplification that integrates thermoelectric heating and ice-valving [20]. However, there remain some outstanding challenges for such systems, including the limited available space in the radial direction, use fabrication technologies that are not compatible with mass production, the chip can't adapt to different reaction system, or the longer PCR reaction time [14,15,16,18,19,20,21,22,23].

In this study, we developed an automated PCR system based on centrifugal microfluidic devices. The novelty in the application comes from the use of a secondary rotational axis to control the positioning of the reaction chamber relative to the centrifugal force, so the sample liquid is bi-directionally transferred through a series of chambers. Moreover, this system can adapt to different reaction system, and the reaction system can programmable flow through three reaction regions at three different temperatures. The process of the PCR disc is easy to realize the industrialization quantity production. We used heating resistors and thermistors to maintain uniform temperatures in each chamber. DNA was quantitated with an optical detection system. Our centrifugal-based PCR disc system was tested by amplifying a target gene of hepatitis B virus (HBV). The results suggest that the system is a promising platform for nucleic acid detection. This system can be used for both DNA amplification and RNA reverse transcription PCR, and suitable for clinical application.

## 2. Materials and Methods

### 2.1. System Design and Construction

**Figure 1:**
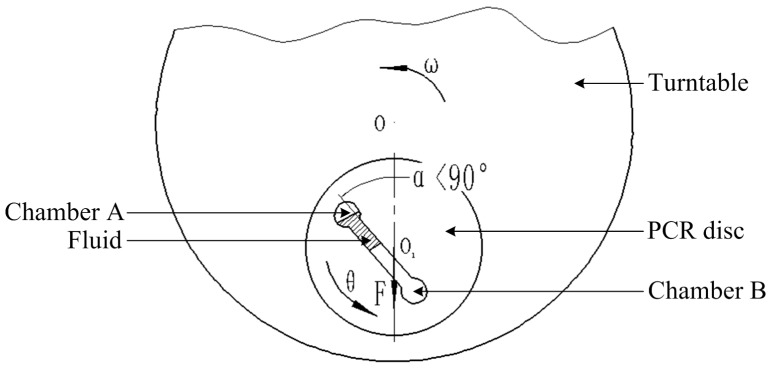
Working diagram of the double-shaft centrifugal microfluidic system. (F: centrifugal force).

In currently used centrifugal microfluidic systems, the fluid moves unidirectionally along the radius to the circumference of the disc under centrifugal force, which limits the utilization of space on the chip. To automate fluid flow and achieve bidirectional flow control, we created a double-shaft centrifugal microfluidic system that included a turntable and PCR disc (Figure 1). In this setup, the turntable rotated around the spindle axis O, and the disc was placed on the turntable on one side of the spindle with a countershaft O_1_ around which the disc rotated at angle θ. The chambers were connected to micro-channels, and the angle θ formed between the latter and the turntable rotation radius changed with angle α of the disc rotating around the countershaft. The spindle and countershaft were driven by servo and stepping motors, respectively. By taking advantage of the relative rotations of the spindle and countershaft, the angle of the micro-channel relative to the spindle rotation radius were adjusted in order to change the size and direction centrifugal force on the liquid with respect to the chip, thereby controlling the movement of the liquid. To transfer the fluid from chambers A to B, the countershaft was first rotated to adjust α to an acute angle between the channel connecting chambers A and B and the turntable rotation radius; the turntable was then rotated at a sufficient speed to allow the fluid in chamber A to flow to chamber B when the centrifugal force of the fluid exceeded the resistance.

**Figure 2:**
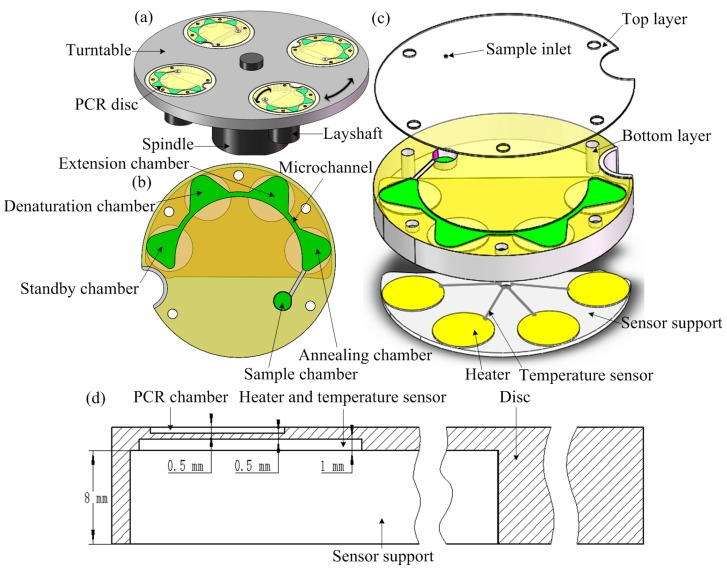
(**a**) Schematic illustration of the fluid control unit consisting of a double-shaft turntable and a centrifugal-based PCR disc; (**b**) Top view of the disc comprising the sample chamber and four reaction chambers; (**c**) Enlarged view of the disc consisting of two layers; (**d**) Section view of the disc.

The centrifugal-based microfluidic system was composed of a fluid control unit and four heat control units, and the overall system comprises four discs in the same turntable which are used simultaneously for replicate experiments (Figure 2a). The fluid control unit included a double-shaft turntable and centrifuge-based PCR disc (Figure 2b) consisting of sample, annealing, extension, and denaturation chambers; A standby chamber for reactions that require four different temperatures and accordingly, four reaction chambers; and some micro-channels. The heat control unit at the back of the PCR unit comprised heating resistors and thermistors that maintained a specific amplification temperature (Figure 2c).

A fluid control unit was used to produce the centrifugal force and pump the fluid through the three chambers in the denaturation, annealing, and extension steps of the PCR. The fluid network is a sealed system that allows the transfer of fluid from one chamber to another when the centrifugal force was large enough to overcome air compression. In addition, a thermal control unit composed of three heating resistors and thermistors made of platinum was placed at the back of the chambers and provided specific amplification temperatures for the three steps of the PCR protocol (Figure 2c). The negative temperature coefficient thermistors were located close to the heating resistors to achieve closed-loop temperature control.

**Figure 3:**
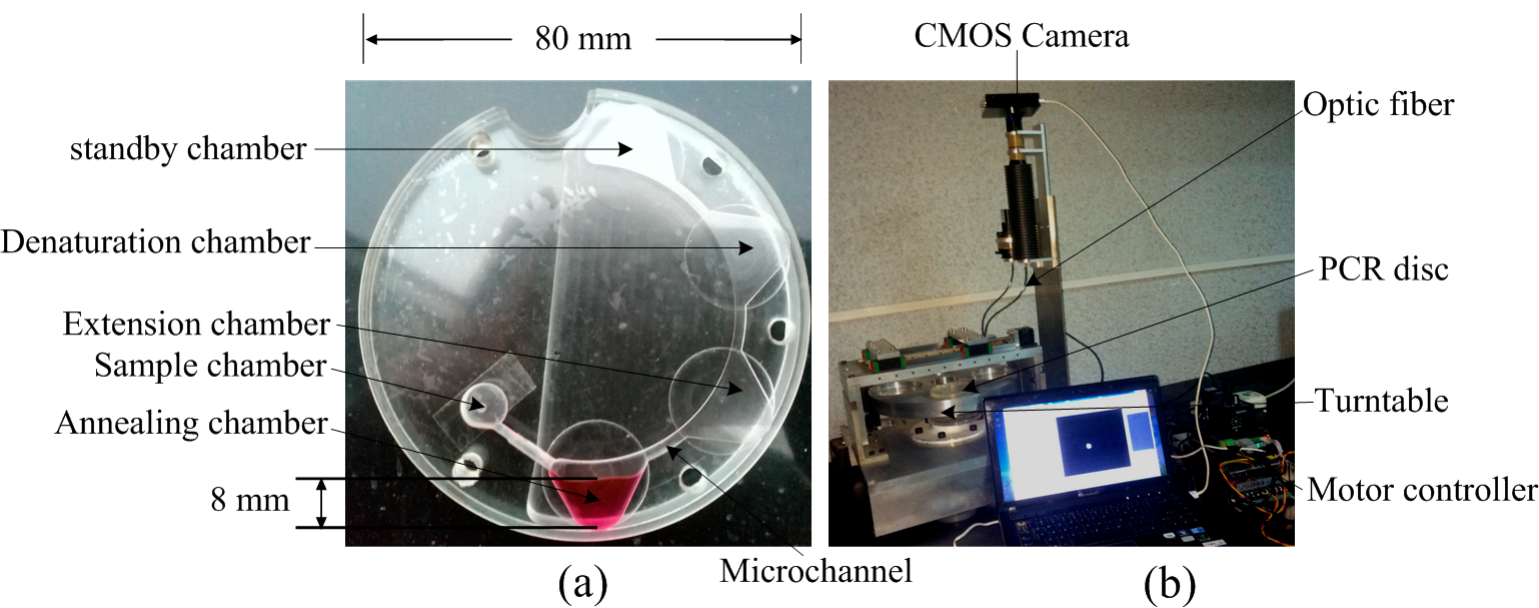
(**a**) Images of an assembled PCR disc; (**b**) The complete system, including turntable, PCR disc, and motor controller.

The centrifugal PCR disc consisted of two polymethyl methacrylate (PMMA) sheets (Figure 2c). The 0.5-mm-thick top layer had a loading hole that was bored with a simple computer numerical control (CNC) instrument (BJD-Y1325ATC; Jindiao, Beijing, China) in which the PCR mixture was sealed using a thin adhesive film to prevent evaporation. The 10-mm thick bottom layer contained the sample and PCR chambers and channels, the back face of the amplification area was engraved 8 mm to mount the sensor support, and 1 mm grooves were engraved to install heater plates using the same CNC machine (Figure 2d). The microfluidic structures were designed using SolidWorks software (Dassault Systèmes SolidWorks Corp., Waltham, MA, USA). The top and bottom layers were bonded together with an adhesive cured by exposure to ultraviolet (UV) light (AiBOND 306; Aibond, Shanghai, China). The depth of the PCR chamber was 0.5 mm, the distance between the resistors and the PCR chambers was 0.5 mm, the section view of the disc was shown in Figure 2d. The diameter of the PCR disc after assembly was 80 mm (Figure 3a).

### 2.2. Experimental Setup

A double-shaft turntable driven by a servo motor and motor controller was used to rapidly pump the fluid through the four PCR chambers. Therefore, the centrifugal microfluidic system for nucleic acid amplification and optical detection consisted of a turntable, PCR disc, motor controller, excitation light source (M470L3-C2; Thorlabs, Newton, NJ, USA), and complementary metal oxide semiconductor (CMOS) camera (C11440-50B; Hamamatsu Photonics, Hamamatsu, Japan) (Figure 3b).

The three heating resistors and thermistors maintained specific temperatures for each of the three steps in the PCR. Before bonding, a thermocouple (wrnk-191; Shanghai Automation Instrumentation, Shanghai, China) was inserted into the PCR chamber for temperature calibration. After bonding the top and bottom layers, the 22-μL PCR mixture was pipetted into the sample chamber, and 18 μL mineral oil were added in order to seal the reaction mixture and prevent fluid evaporation. The temperature distribution of the three chambers was measured with an infrared (IR) camera (R300; NEC, Kanazawa, Japan).

This centrifugal microfluidic system also includes a real-time fluorescent detection module, an excitation light source and CMOS camera were used for excitation and fluorescence signal detection, respectively (Figure 3b). A blue light-emitting diode with peak emission at 470 nm was used as the excitation light source; an optic fiber fixed in the framework detected the signal when the chamber on the turntable rotated into position. Fluorescence intensity changes during thermal cycling were used to determine the threshold cycle (C_t_) value, which was defined as the cycle number at which the fluorescence signal is equivalent to the background level up to the exponential growth phase. The amplification efficiency (E_ff_) depended on the rate of the standard curve, which was generated based on Ct values and target DNA concentration. E_ff_ was calculated with the following equation [24]:

E_ff_ = 10^(−1/slope)^ (1)

The reaction mixture was transferred from the sample chamber to the denaturation chamber, and then pumped to the annealing chamber after completion of the pre-denaturation and denaturation steps. Fluorescence was detected when the reaction mixture was moved to the extension chamber for the elongation step. The PCR mixture was sequentially moved through these three chambers during cycling. The fluid flow rate was determined by measuring the time required to transfer 40 μL of water at various motor operating frequencies of the turntable. An average flow rate was determined from three experiments.

### 2.3. PCR Reagents

To assess the functioning of the PCR disc, a target gene of hepatitis B virus (HBV) DNA with a length of 350 base pairs (bp) was amplified using the forward and reverse primers 5′-CCGATCCATACTGCGGAAC-3′ and 5′-GCAGAGGTGAAGCGAAGTGCA-3′, respectively. The 22-μL reaction mixture contained 2 μL SYBR Green I labeling dye, 6.6 μL double distilled H_2_O (ddH_2_O), 2.4 μL of 25 mM Mg^2+^, 2 μL each of 2 mol forward and reverse primers, 2 μL Taq polymerase, and 5 μL HBV DNA template at different initial concentrations (10–10^6^ copies/mL) [25].

The thermal cycling protocol for amplification of the HBV gene was as follows: pre-denaturation at 94 °C for 10 min; 40 cycles of denaturation at 94 °C for 30 s, annealing at 53 °C for 30 s, and extension at 72 °C for 40 s; and terminal elongation at 72 °C for 4 min. This is a standard procedure for benchtop PCR instruments, and the cycling time includes temperature rise/fall, soaking, thermal equilibrium, and detection time. The system did not require heating/cooling time, and the detection time of 1 s was much faster than the time of 15 s on a commercial PCR instrument. A series of amplification reactions with different cycling time was carried out to determine the optimal parameters of this system.

To prevent evaporation of the reaction mixture, 18 μL mineral oil (Amresco, Solon, OH, USA) were injected along with the PCR mixture into the sample chamber. The oil was added to prevent evaporation by forming a top layer over the PCR reaction mixture. When the oil and water-based PCR mixture was transferred to one chamber under the effect of centrifugal force, the centrifugal force caused the chamber to be filled from the outer wall to the center of the disc, the oil is closer to the center relative to the water-based PCR mixture because the density of oil is less than water. When the amplification was completed, both the reaction mixture and mineral oil were draw out of the chamber.

The amplified product was visualized by electrophoresis on a 2% agarose gel stained with Goldview (Viswagen Biotech, Kerala, India). A DNA marker (DL2000; Takara Bio, Dalian, China) was to estimate product size and the gel was imaged by exposure to UV light. DNA was also amplified in parallel on a commercial benchtop PCR instrument (TL988; Tianlong, Xi’an, China) to assess the relative amplification rate and efficiency of the centrifugal microfluidic system.

### 2.4. Experimental Procedure

The 22-μL PCR mixture and 18-μL mineral oil were loaded into the sample chamber with a pipettor and adhesive tape was used to seal the sample hole to prevent evaporation. The PCR disc was mounted on the turntable, and the annealing chamber was positioned farthest from the spindle relative to other chambers (Figure 5a). Three heating resistors and thermistors were in contact with the back face of the chamber and maintained temperatures of 94 °C, 72 °C, and 53 °C in the denaturation, extension, and annealing chambers, respectively. The disc was then rotated as described below.

The spindle was first fixed in place and the PCR disc was rotated to put the denaturation chamber in the farthest from the spindle relative to other chambers (Figure 5b); The countershaft was first fixed and the turntable was spun clockwise for 4 s at 15 Hz to move the 40-μL reaction mixture into the denaturation chamber, which was heated at 94 °C for 10 min (Figure 5b). This was followed by initial denaturation at 94°C. The spindle was fixed in place and the PCR disc was rotated to put the annealing chamber in the farthest from the spindle relative to other chambers (Figure 5c); the countershaft was then immobilized and the turntable was spun clockwise for 4 s at 15 Hz to move the 40-μL reaction to the annealing chamber where it was incubated at 53°C (Figure 5c). The spindle was immobilized, and the PCR disc was rotated to put the extension chamber in the farthest from the spindle relative to other chambers (Figure 5d); The countershaft was then fixed in place and the turntable was spun counterclockwise for 2 s at 15 Hz to move the 40-μL reaction to the extension chamber where it was heated at 72 °C (Figure 5d). The three steps (denaturation, annealing, and extension) were repeated 40 times before the sample was heated at 72 °C for 4 min for terminal extension. The total amplification time was about 80 min; the fluidic operation time was discounted, and the biological reaction time was decided by the reagents.

When cycling was complete, the spindle was immobilized and the PCR disc was rotated to put the sample chamber in the farthest from the spindle relative to other chambers, thus the amplified products and mineral oil in the extension chamber were moved to the sample chamber of the disc, and then transferred by pipettor from the sample hole and stored at 4 °C until further processing.

## 3. Results and Discussion

### 3.1. PCR Chamber Design and Fluid Control

When liquid was moved between chambers, the centrifugal force caused the chambers to be filled from the outer wall to the center. The chamber had depths of 0.5 mm, and their uniform design allowed improved heat transfer owing to increased surface area.

**Figure 4:**
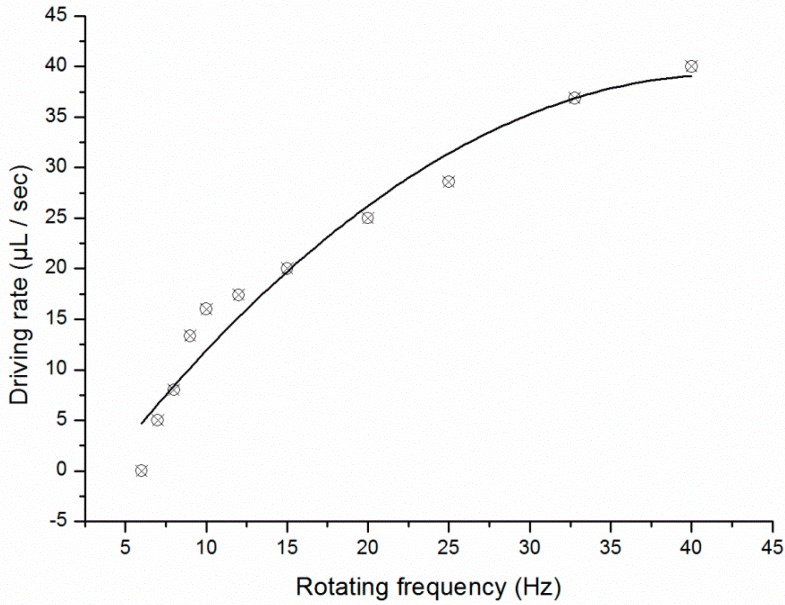
Relationship between sample flow rate and rotating frequency of the fluid control unit.

A double-shaft turntable was used to transfer the PCR mixture. The rotating frequency of the servo motor controlled the time between each rotation of the turntable. Fluid flow rate increased as a function of turntable rotation frequency (Figure 4); a typical flow rate of 20 μL/s was achieved at a rotating frequency of 15 Hz which is the optimized parameter on our setup.

**Figure 5:**
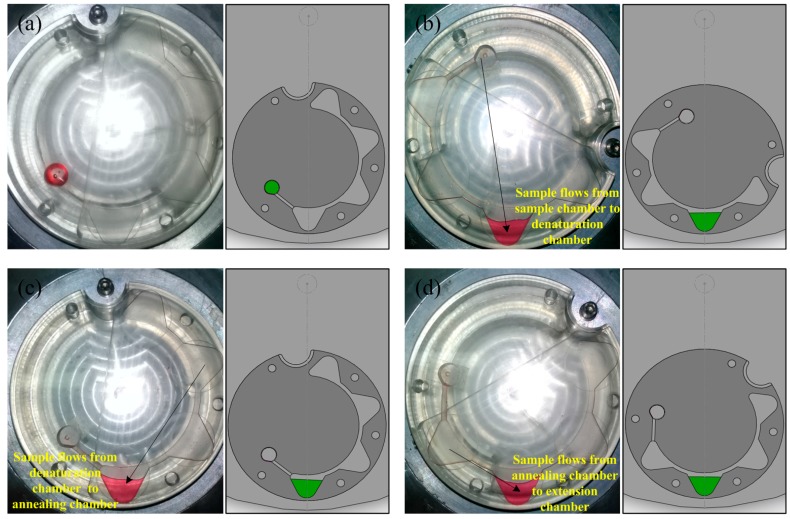
Images and schematics of fluid flow through the three PCR chambers. (**a**) The initial position of the PCR disc; (**b**) 40-μL reaction mixture was pumped to the denaturation chamber from the sample chamber; (**c**) 40-μL reaction mixture was pumped to the annealing chamber from the denaturation chamber; (**d**) 40-μL reaction mixture was pumped to the extension chamber from the annealing chamber.

40 μL of water was transferred from one chamber to another to verify the fluid can flow through these PCR chambers of the disc by using the fluid control unit (Figure 5). The cycle times and reaction time of the three PCR steps could be independently adjusted. Moreover, residual PCR mixture that normally constitutes a dead volume in the PCR chamber and can affect the efficiency of amplification [25] was eliminated due to rapid transfer of the reaction mixture by the double-shaft turntable.

Although a constant temperature was maintained in each of the three chambers, the PCR mixture requires a certain amount of time to achieve thermal balance when transferred from one chamber to another. In this study, about 2 s was required for the 22-μL reaction mixture to attain this state.

### 3.2. Temperature Control

The ability to maintain a specific temperature is especially important for PCR; the efficiency of the amplification can be affected by the temperature distribution within the PCR chamber, with a non-uniform temperature creating a thermal gradient that can lead to the production of non-specific products at the annealing stage. In this study, heating resistors with closed-loop control function were used to enhance the uniformity of the temperature field in the PCR chambers.

**Figure 6:**
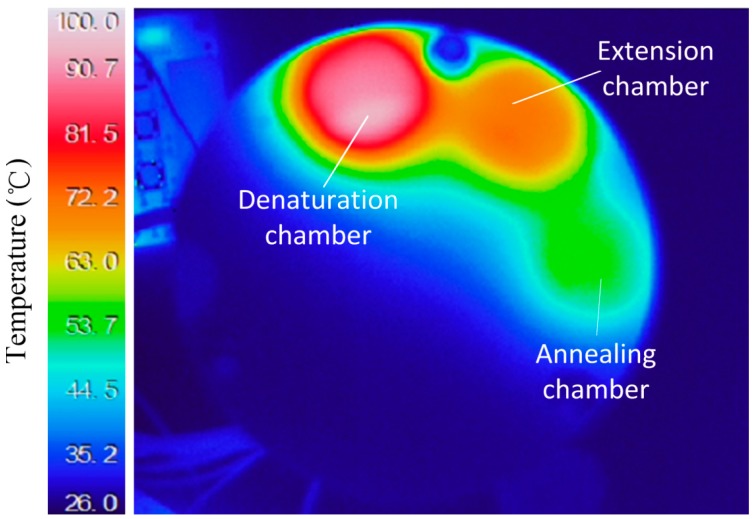
IR image of temperature fields in the three PCR chambers.

Three heating resistors and thermistors provided driving currents that maintained specific temperatures at each of the three steps of the PCR. The thermistors were located close to the resistors and used for signal transmission and feedback. Temperature distributions of the three (empty) PCR chambers are shown in Figure 6; the IR image shows the surface temperatures. When the oil and water-based PCR mixture was loaded, the PCR mixture can preferably absorb the heat from the heater because water has a higher specific heat than air, so the temperature distributions were more uniform after the PCR mixture was added; the thermal variation in the chambers was <2 °C, which was sufficiently small for most amplifications [26]. Moreover, there was little thermal interference between the three chambers.

### 3.3. DNA Amplification and Quantification 

The 350-bp product corresponding to the HBV gene at different initial concentrations (10–10^6^ copies/mL) was amplified on both a commercial benchtop PCR machine and the PCR disc. The fluorescence emission at 520 nm was detected for 1 s at the extension stage, which had a reaction temperature of 72 °C. The fluorescence intensity of water, which was measured as 1.8 arbitrary units, was used as a reference.

The minimum cycling time that could be obtained with our system was determined via a series of amplification reactions with different cycling time. C_t_ values (mean ± SD) using 10^3^ copies/mL DNA were determined on a commercial PCR machine (TL 988) and our centrifugal microfluidic PCR chip with the standard PCR procedure (the denaturation, annealing and extension times were 30 s, 30 s and 40 s). The C_t_ values of the TL 988 and the PCR disc were 32.1 ± 0.42 and 32.3 ± 0.50. The discrepancy between the C_t_ values may be explained by inaccuracies and temperature non-uniformity in our system as compared to the commercial apparatus. Using the centrifugal microfluidic PCR chip, similar C_t_ values (mean ± SD) were obtained with PCR protocols in which the denaturation time was modified (denaturation times of 10, 5, 3 and 2 s, respectively, with annealing and extension times of 30 and 40 s, respectively, as shown in Table 1), a greater variability was observed for 2 s than for 10s, 5 s or 3 s, suggesting that a denaturation time of 3 s is adequate for this system. When annealing time was modified (annealing time of 15, 10, and 9 s, respectively, with denaturation and extension time of 30 and 40 s, respectively, as shown in Table 2), a greater variability was observed for 9 s than for 15 s or 10 s, suggesting that an annealing time of 10 s is suitable for this system. When extension time was modified (extension time of 15, 10, 5, and 4 s, respectively, with denaturation and annealing time of 30 s, respectively, as shown in Table 3), a greater variability was observed for 4 s than for 15s, 10 s or 5 s, suggesting that an extension time of 5 s is appropriate for this system. Therefore, according to the above experimental results, thermal cycling in the centrifugal microfluidic system can consist of 3-s denaturation, 10-s annealing, and 5-s extension steps, which would yield a total amplification time of about 32 min, roughly 27% of the time required using a benchtop machine (120 min).

**Table 1:** C_t_ values with denaturation time of 10, 5, 3 and 2 s, respectively, annealing and extension time of 30 and 40 s, respectively.

	Denaturation Time (s)			
	10	5	3	2
**C_t_ (Mean ± SD)**	32.2 ± 0.48	32.3 ± 0.49	32.3 ± 0.48	33.4 ± 0.72

**Table 2:** C_t_ values with annealing time of 15, 10, and 9 s, respectively, denaturation and extension time of 30 and 40 s, respectively.

	Annealing Time (s)		
	15	10	9
**C_t_ (Mean ± SD)**	32.2 ± 0.49	32.3 ± 0.51	32.9 ± 0.68

**Table 3:** C_t_ values extension time of 15, 10, 5, and 4 s, respectively, with denaturation and annealing time of 30 s, respectively.

	Extension Time (s)			
	15	10	5	4
**C_t_ (Mean ± SD)**	32.2 ± 0.48	32.3 ± 0.50	32.3 ± 0.51	33.2 ± 0.67

**Figure 7:**
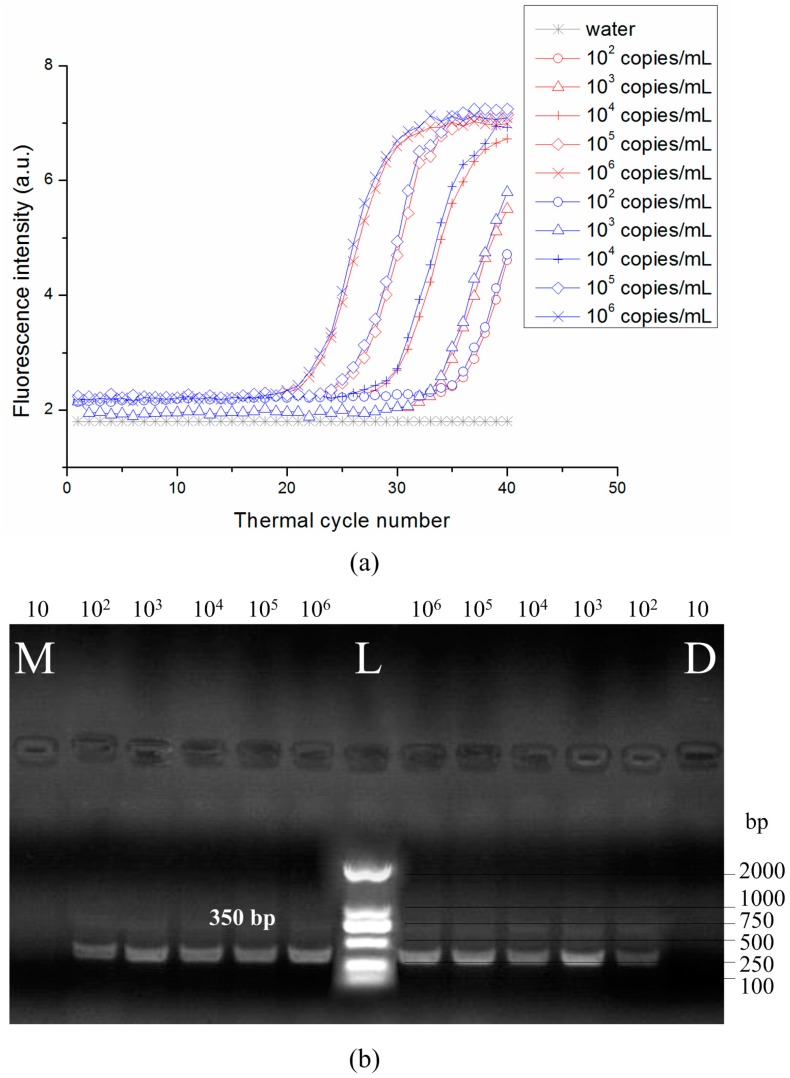
The PCR was conducted under with the optimized PCR protocol (3-s denaturation, 10-s annealing and 5-s extension). (**a**) Fluorescence intensity *vs.* cycle number for the detection of HBV gene (350 bp) with known initial concentrations of template (10^2^–10^6^ copies/mL) (blue lines: commercial PCR machine; red lines: disc PCR); (**b**) Gel electropherograms for detection of HBV and template with known initial concentrations (10–10^6^ copies/mL). (L: DNA marker; M: commercial PCR machine; D: disc PCR.)

The fluorescence intensity curve for different initial concentrations of template (10–10^6^ copies/mL) with the optimized PCR protocol is shown in Figure 7a. The fluorescence signal in the amplification reaction with 10 copies/mL DNA template was <1.8 arbitrary units, and it was therefore omitted from the curve. Amplified products were also analyzed by gel electrophoresis (Figure 7b).

**Figure 8:**
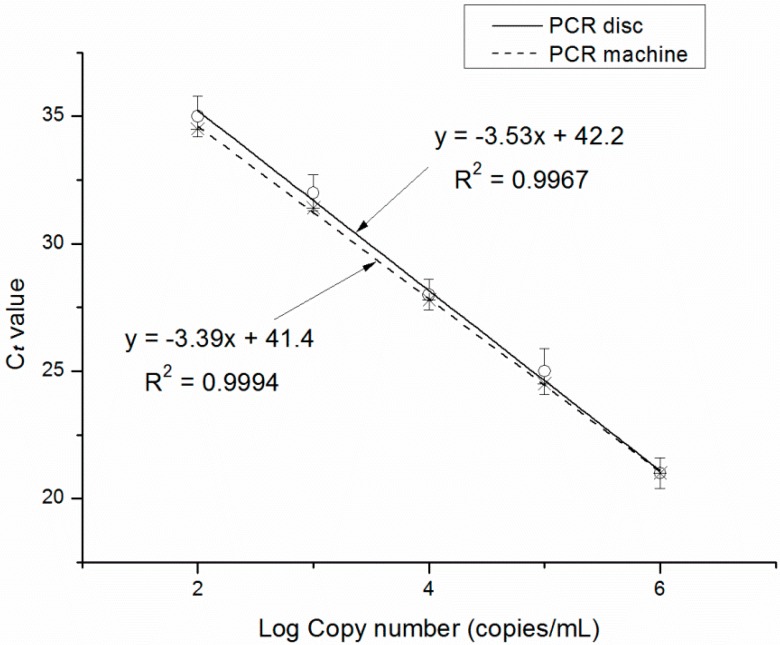
Standard curve for HBV target gene amplification.

**Figure 9:**
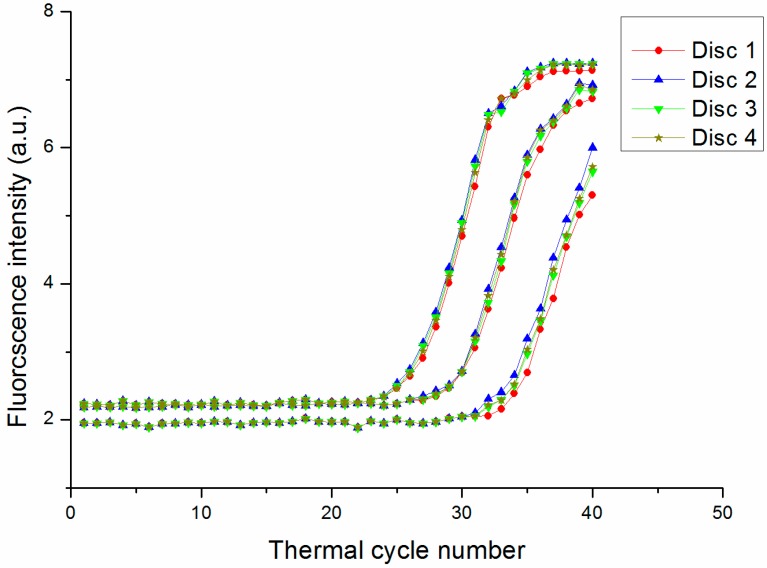
The fluorescence intensity curve for 10^3^, 10^4^ and 10^5^ copies/mL DNA with four discs in the same turntable simultaneously.

Imprecision of the optical detection system as well as non-uniform thermal cycling can affect the reproducibility of the PCR protocol [27]; this along with amplification efficiency is determined by the C_t_ value and standard curves. We observed that a high initial DNA concentration resulted in an earlier time point for the exponential increase in signal intensity (Figure 7a). In the standard curve for the HBV gene, C_t_ value decreased with increasing concentration (Figure 8). E_ff_ was 1.92 and 1.97 for the PCR disc and commercial machine, respectively. The E_ff_ of 1.92 is high enough to validate the availability of the PCR disc although the E_ff_ is lower than commercial machine. The lower value for the PCR disc may be due to the fact that optical detection and/or temperature control were not optimized in our system. In addition, the four discs in the turntable of the overall system were used to amplify HBV-DNA gene simultaneously. The fluorescence intensity curve for 10^3^, 10^4^ and 10^5^ copies/mL DNA with the optimized PCR protocol is shown in Figure 9. The fluorescence intensity curve showed good consistence of the four discs.

## 4. Conclusions

This study describes the development of an automated centrifugal-based PCR disc that uses a double-shaft turntable to pump liquid through three independent reaction chambers and the bidirectional flow reaches the maximum usable area of the disc, the PCR disc can fit reaction systems from 5 μL to 40 μL, and the manufacturing process of the PCR disc should be easy to realize in industrial quantities. A flow rate of 20 μL/s was achieved at a turntable rotating frequency of 15 Hz. Three heating resistors and thermistors with a closed-loop control feature were used to maintain the different temperatures of a PCR program and provided a homogeneous temperature distribution within each chamber. An optical detection system was used to quantify the amplified products of HBV-DNA gene; the quantitative detection limit was 10^2^ copies/mL, and amplification time was reduced to 32 min as compared to 120 min for a commercial instrument. The design of this centrifugal-based microfluidic platform—which integrates pumping, thermocycling, and detection—makes it a useful system and it possesses industrialization potential for portable diagnostics. Future work will integrate other steps of nucleic acid analysis like sample preparation and nucleic acid purification in the remainding space on the same disc, optimize the optical detection and/or temperature control modules, and use the system for nested PCR and DNA hybridization.

We aim at integrating the sample preparation module on this disc, so in the future it will constitute a sample-to-answer system. We have estimated the cost of the disposable disc to be about $3. We expect that this sensor can be actually used for on-field analysis, although a possible limitation is the size because this system includes a spindle and countershaft. Nevertheless, the disc is low cost product, with easy operation, that can be used for clinical detection.

## References

[1] Lin Y.C., Huang M.Y., Young K.C., Chang T.T., Wu C.Y. (2000). A rapid micro-polymerase chain reaction system for hepatitis C virus amplification. Sens. Actuators B Chem..

[2] Lee D.S., Park S.H., Yang H., Chung K.H., Yoon T.H., Kim S.J., Kim K., Kim Y.T. (2004). Bulk-micromachined submicroliter-volume PCR chip with very rapid thermal response and low power consumption. Lab Chip.

[3] Liu L.Y., Cao W.B., Wu J.B., Wen W.J., Chang D.C., Sheng P. (2008). Design and integration of an all-in-one biomicrofluidic chip. Biomicrofluidics.

[4] Qiu X.B., Michael G.M., Chen D.F., Liu C.C., Haim H.B. (2010). A large volume, portable, real-time PCR reactor. Lab Chip.

[5] Yoon D.S., Lee Y.S., Lee Y., Cho H.J., Sung S.W., Oh K.W., Cha J., Lim G. (2002). Precise temperature control and rapid thermal cycling in a micromachined DNA polymerase chain reaction chip. Micromech. Microeng..

[6] Utsumi Y., Hitaka Y., Matsui K., Takeo M., Negoro S., Ukita Y. (2007). Planar microreactor for biochemical application made from silicon and polymer films. Microsyst. Technol..

[7] Kopp M.U., Manz A. (1998). Chemical amplification: Continuous-flow PCR on a chip. Science.

[8] Qi H., Wang X.S., Chen T., Ma X.M., Zuo T.C. (2009). Fabrication and characterization of a polymethyl methacrylate continuous-flow PCR microfluidic chip using CO_2_ laser ablation. Microsyst. Technol..

[9] Fang T.H., Ramalingam N., Xian-Dui D., Ngin T.S., Xianting Z., Kuan A.T.L., Hai-Qing G. (2009). Real-time PCR microfluidic devices with concurrent electrochemical detection. Biosens. Bioelectron..

[10] Nagatani N., Yamanaka K., Ushijima H., Koketsu R., Sasaki T., Ikuta K., Tamiya E. (2012). Detection of influenza virus using a lateral flow immunoassay for amplified DNA by a microfluidic RT-PCR chip. Analyst.

[11] Nie J., Zhao Y., Peng N. (2014). Multichannel oscillatory-flow PCR micro-fluidic chip with controllable temperature gradient. Microsyst. Technol..

[12] Gransee R., Schneider T., Elyorgun D., Strobach X., Schunck T., Gatscha T., Höth J. Fluorescence detection in Lab-on-a-chip systems using ultrafast nucleic acid amplification methods. Proceedings of the Smart Biomedical and Physiological Sensor Technology XI.

[13] Almassian D.R., Cockrell L.M., Nelson W.M. (2013). Portable nucleic acid thermocyclers. Chem. Soc. Rev..

[14] Madou M., Zoval J., Jia G., Kido H., Kim J., Kim N. (2006). Lab on a CD. Annu. Rev. Biomed. Eng..

[15] Gorkin R., Park J., Siegrist J., Amasia M., Lee B.S., Park J.M., Cho Y.K. (2010). Centrifugal microfluidics for biomedical applications. Lab Chip.

[16] Ducree J., Haeberle S., Lutz S., Pausch S., Stetten F.V., Zengerle R. (2007). The centrifugal microfluidic Bio-Disk platform. Micromech. Microeng..

[17] Burger R., Kirby D., Glynn M., Nwankire C., O’Sullivan M., Siegrist J., Kinahan D., Aguirre G., Kijanka G., Gorkin R.A. (2012). Centrifugal microfluidics for cell analysis. Curr. Opin. Chem. Biol..

[18] Strohmeier O., Emperle A., Roth G., Mark D., Zengerle R., Stetten F. (2013). Centrifugal gas-phase transition magnetophoresis (GTM)—A generic method for automation of magnetic bead based assays on the centrifugal microfluidic platform and application to DNA purification. Lab Chip.

[19] Wang G., Ho H.P., Chen Q., Yang A.K.L., Kwok H.C., Wu S.Y., Zhang X. (2013). A lab-in-a-droplet bioassay strategy for centrifugal microfluidics with density difference pumping, power to disc and bidirectional flow control. Lab Chip.

[20] Amasia M., Cozzens M., Madou M. (2012). Centrifugal microfluidic platform for rapid PCR amplification using integrated thermoelectric heating and ice-valving. Sens. Actuators B Chem..

[21] Kellogg G.J., Arnold T.E., Carvalho B.L., Duffy D.C., Sheppard N.F. (2000). Centrifugal microfluidics: Applications. Micro Total Analysis Systems 2000.

[22] Park B.H., Jung J.H., Zhang H., Lee N.Y., Seo T.S. (2012). A rotary microsystem for simple, rapid and automatic RNA purification. Lab Chip.

[23] Strohmeier O., Keller M., Schwemmer F., Zehnle S., Mark D., von Stetten F., Zengerle R., Paust N. (2015). Centrifugal microfluidic platforms: Advanced unit operations and applications. Chem. Soc. Rev..

[24] Pfaffl M.W. (2001). A new mathematical model for relative quantification in real-time RT–PCR. Nucl. Acids Res..

[25] Chien L.J., Wang J.H., Hsieh T.M., Chen P.H., Chen P.J., Lee D.S., Lee G.B. (2009). A micro circulating PCR chip using a suction-type membrane for fluidic transport. Biomed. Microdev..

[26] Hsieh T.M., Luo C.H., Huang F.C., Wang J.H., Chien L.J., Lee G.B. (2008). Enhancement of thermal uniformity for a microthermal cycler and its application for polymerase chain reaction. Sens. Actuators B Chem..

[27] Wang J.H., Chien L.J., Hsieh T.M., Luo C.H., Chou W.P., Chen P.H., Lee G.B. (2009). A miniaturized quantitative polymerase chain reaction system for DNA amplification and detection. Sens. Actuators B Chem..

